# Pancreatic Ductal Adenocarcinoma Cortical Mechanics and Clinical Implications

**DOI:** 10.3389/fonc.2022.809179

**Published:** 2022-01-31

**Authors:** Shantel Angstadt, Qingfeng Zhu, Elizabeth M. Jaffee, Douglas N. Robinson, Robert A. Anders

**Affiliations:** ^1^Department of Pathology Johns Hopkins University School of Medicine, Baltimore, MD, United States; ^2^Department of Oncology, Johns Hopkins University School of Medicine, Baltimore, MD, United States; ^3^Department of Cell Biology, Johns Hopkins University School of Medicine, Baltimore, MD, United States

**Keywords:** PDAC, cytoskeleton, cortical mechanics, cell shape, clinical implications

## Abstract

Pancreatic ductal adenocarcinoma (PDAC) remains one of the deadliest cancers due to low therapeutic response rates and poor prognoses. Majority of patients present with symptoms post metastatic spread, which contributes to its overall lethality as the 4th leading cause of cancer-related deaths. Therapeutic approaches thus far target only one or two of the cancer specific hallmarks, such as high proliferation rate, apoptotic evasion, or immune evasion. Recent genomic discoveries reveal that genetic heterogeneity, early micrometastases, and an immunosuppressive tumor microenvironment contribute to the inefficacy of current standard treatments and specific molecular-targeted therapies. To effectively combat cancers like PDAC, we need an innovative approach that can simultaneously impact the multiple hallmarks driving cancer progression. Here, we present the mechanical properties generated by the cell’s cortical cytoskeleton, with a spotlight on PDAC, as an ideal therapeutic target that can concurrently attack multiple systems driving cancer. We start with an introduction to cancer cell mechanics and PDAC followed by a compilation of studies connecting the cortical cytoskeleton and mechanical properties to proliferation, metastasis, immune cell interactions, cancer cell stemness, and/or metabolism. We further elaborate on the implications of these findings in disease progression, therapeutic resistance, and clinical relapse. Manipulation of the cancer cell’s mechanical system has already been shown to prevent metastasis in preclinical models, but it has greater potential for target exploration since it is a foundational property of the cell that regulates various oncogenic behaviors.

## Introduction

### Cell Mechanics and Cancer

Cell mechanics refers to the cell’s physical properties and the mechanisms of force detection, force production, and load bearing to generate cell shape and behavior. More broadly, cell mechanics can encompass the application of solid and fluid concepts from physics and engineering to cells and the larger structures they compose (1, 2). To perform essential functions, such as tissue development, cell division, apoptosis, and migration, cells use internal and external stimuli to drive cell shape change and other highly mechanical processes. Morphogenesis results from the rearrangement of the cell’s underlying cytoskeleton, which changes the physical properties of the cell. Cytoskeletal rearrangement and the resulting cell shape change require active force generation initiated by the integration of chemical and mechanical (mechanochemical) signals. Understanding processes involving cell shape modification will lend new insights for the treatment of pathological states resulting from dysfunctions in cell division, apoptosis, and migration, *i.e.* cancer transformation and metastasis (3, 4).

The cortical cytoskeleton is responsible for cell shape change and is primarily composed of actin filaments, crosslinking proteins, and nonmuscle myosin II (NMII) filaments. The various regulators of these proteins are also vital contributors to morphogenesis since they control the spatiotemporal assembly and disassembly of cytoskeletal filaments. Actin filaments’ semi-flexible property, in addition to actin crosslinkers, allow for the interconnected network of filaments to form the cortical cytoskeleton and propagate mechanical stresses around the entire cell. The molecular properties and resulting function of the three components culminate into the mechanical properties of cell behavior. For example, cooperative interactions between NMII and crosslinking proteins allow these proteins to accumulate at sites of stress along the cell cortex. Overall cell shape change is managed through the integration of actin filament turnover, actin crosslinking, and NMII contractility and cooperativity (5–9).

The molecular binding affinities of the structural proteins with each other allow the cytoskeleton to maintain a fixed structure and resist deformation on short time scales, behaving elastically like a solid material. Likewise, these same binding affinities and regulatory mechanisms allow for protein disassociation and cytoskeletal rearrangement lending the cells viscous behavior, like a liquid, on longer timescales. Therefore, the cell is defined mechanically as a viscoelastic material often represented by both elastic spring and viscous damper components in mathematical models. Modeling can help predict cell behaviors, which is an invaluable tool for understanding cell processes such as cytokinesis and motility (8, 10, 11).

A major focus of the field has been to characterize mechanical properties that regulate cell and tissue function, especially in disease states. The ultimate goal is to use mechanical properties to generate new perspectives for various diseases and corresponding prognoses and treatments. Researchers have proposed a structure-property-function-disease paradigm in investigating the mechanics of cancer transformation and progression. For example, investigating the signaling effectors leading to physical properties of cells, how these physical properties lend function, and how the dysfunction at any part (signaling molecules, physical properties, resulting function) leads to diseased states. But cell mechanics is a complex and multiscale field: properties at the molecular, cellular, and tissue levels must each be characterized as well as integrated with the dynamic feedback systems. In the specific case of tumor tissue, the actin cytoskeleton at the molecular level generates the mechanical properties of individual cells, and the tissue level mechanical properties (*i.e.*, stiffness) of the tumor feedback into individual cell’s mechanics resulting in altered function (differentiation, proliferation, invasion). Although complex, an understanding of tumor cell mechanics and the metastatic process opens an entirely new field for prognoses and therapeutic targeting (1, 2, 7, 8, 12).

Moreover, mechanical microenvironments in tumors are transformed along with the individual tumor cells. For example, the extracellular matrix (ECM) of the tumor is often stiffer than the surrounding healthy tissue. Tumor cells have been shown to have more invasive phenotype on stiffer substrates and this phenotype can be reversed with substrate tension relaxation. There is evidence that tumor cells soften in response to this stiffening ECM. Additionally, tumor tissues experience increased fluid pressures due to angiogenesis and tissue restructuring. The altered mechanical states of the tumor tissue initiate mechanosensing in individual tumor cells. Mechanosensing pathways have been shown to drive proliferation, survival, invasion, stemness, and therapy resistance. Therefore, mechanical stimuli and their effects are multiscale: tissue-level, cell-to-cell interaction, cell-to-matrix interactions, and biochemical reactions (13).

In addition to the ECM altering tumor cell function through mechanosensing, tumor cells have the ability to remodel the ECM and the polarity of cancer-associated fibroblasts and different immune cells. For example, another focus of cancer cell mechanics has been traction forces and polarity generated by tumor cell contractility during adhesion and migration. Understanding cell-generated traction forces is necessary because these stresses help restructure the ECM and push cells forward during migration. A correlation exists between traction forces, contractility, and metastatic potential. For example, metastatic cells across three cancer types exerted greater traction forces in response to matrix stiffness compared to their non-metastatic counterparts (14–16). These highly metastatic cells with larger traction forces lead the way for collective migration by restructuring collagen fiber alignment into tracks that others cells could more easily follow. Cell polarity also plays a role in mesenchymal modes of cell migration where the direction and persistence of migration is dictated by the alignment of cell and matrix-remodeling polarity (17, 18). Additionally, cytoskeletal forces are physically transmitted to the nucleus through the Linker of Nucleoskeleton and Cytoskeleton (LINC) complex. For example, substrate stiffness can lead to alterations in nuclear stress and shape, resulting in changes of gene expression, nuclear stiffness, and the cell’s differentiation state (19–21).

Furthermore, the metastatic cascade is a physical and mechanically-driven process. Metastasis is both the process and result of cancer cells migrating and colonizing in a location other than the primary tumor site. First, tumor cells must break adhesion complexes with their surrounding cells and migrate through tumor stroma. This migration involves deformation to squeeze through ECM pores, push and pull ECM fibers, and degrade fibers. Second, the tumor cell must invade through a basement membrane and vasculature wall for intravasation, which requires continued matrix degradation and shape deformation. Third, tumor cells in circulation must resist shear forces inflicted by blood flow and adhere to the vessel wall for extravasation. Extravasation involves another deformation process followed by migration/invasion into the new tissue site. Finally, tumor cells must initiate proliferation to colonize the metastatic tumor (22).

Cell deformability is essential for the metastatic cascade and is dependent on the cell’s viscoelasticity. Another major focus within cancer cell mechanics is on the viscoelastic differences between normal, transformed, and metastatic cells. The aim is to characterize the relationship between deformability and metastatic potential. Using optical stretching and atomic force microscopy, several studies across different cancer types revealed that cell deformability increases with metastatic potential. Interestingly, cells can actively resist externally-imposed deformation by polymerizing actin and recruiting specific cytoskeletal proteins to increase tension. It is the dynamic ability of being able to deform and simultaneously resist deformation that allows tumor cells to undergo the metastatic cascade. Therefore, a method to prevent metastasis for solid tumors would be to stiffen and decrease the cell shape change ability (8, 13, 23–27).

Cell and tissue deformation mechanics during cancer transformation and progression is a relatively young field in cancer biology and is working on uncovering a different system of therapeutic targets to address metastasis and patient mortality. Cell deformability and morphogenesis are foundational components of signaling networks, division, adhesion, migration, invasion, and metastatic potential. The shape and rigidity of a cell is due to the cytoskeleton and its molecular components. Previous reports have shown altered cytoskeletal structure, regulation pathways, and extracellular matrix structures in various cancer types and stages. Additionally, metrics of mechanical properties correlate with disease state and metastatic potential. Targeting the cytoskeleton has already been shown to alter mechanical properties and metastasis in preclinical models and is being investigated as an indicator for disease stage and prognoses (26–32). The major challenge in deformation mechanics is that the cell cannot be fully characterized by the static mechanical properties of solids and fluids. Cells are dynamic systems that react and respond to internal and external stimuli. Therefore, their subcellular and physical properties are constantly changing (33). We have already seen the potential of altering the cell’s physical properties to prevent cancer progression. Therefore, a critical need exists to fully integrate the field of cell mechanics in cancer therapeutic and prognostic development.

## PDAC and PDAC-Specific Cortical Mechanics

Pancreatic ductal adenocarcinoma (PDAC) is the most common type of pancreatic cancer. Pancreatic cancer incidence continues to increase across the country in conjunction with it having one of the lowest 5-year relative survival rates around 10%. Patients typically present with symptoms post metastatic spread, which contributes to its overall lethality as the 4^th^ leading cause of cancer-related deaths (34, 35). Advancements in therapy and precision medicine have helped to increase the low 5-year survival rate, but recent discoveries have also uncovered how little we understand PDAC transformation and progression. Our lack of knowledge for the PDAC-specific pathogenesis of transformation and metastasis limits our ability to innovate more effective treatments.

PDAC forms from precursor lesions and has historically presented as a genetic disease, gradually progressing through a sequence of acquired mutations. Four driver genes have been linked to each stage of transformation (*KRAS*, *TP53*, *SMAD4*, and *CDKN2A*), but recurrent somatic mutations (SNV, indel, scNA) and germline mutations (in DNA damage repair genes) have also been found. PDAC is characterized by desmoplastic reaction due to interactions between cancer, vasculature, pancreatic stellate, and inflammatory cells. Over half of all cases are diagnosed post-metastatic spread, and the most common sites for metastasis are stomach, lung, colorectum, esophagus, gall bladder, liver, and common bile duct (2, 36, 37).

The standard treatment is surgical resection and/or two chemotherapeutic agents, gemcitabine and FOLFIRINOX (the combination of oxaliplatin, irinotecan, fluorouracil, and leucovorin) depending on disease severity and stage, but these treatments are least effective post metastatic spread (38, 39). Novel whole genome sequencing and bioinformatic techniques on primary and metastatic tumor clonal populations have revealed that not all PDAC tumors progress through the gradual sequence of transformation steps. Additionally, some PDAC tumors result from a more prolonged precursor lesion stage and show micrometastasis early in the tumor formation process (5, 40–44). Altogether, recent genomic discoveries reveal that genetic heterogeneity and early micrometastases within PDAC progression result in the inefficacy of current standard treatments and specific molecular targeted therapies.

The PDAC tumor microenvironment (TME) is a major contributor to inefficacious treatments, especially for immunotherapies. The PDAC TME is characterized by dense stroma and a small cell content composed of tumor cells, cancer-associated fibroblasts, muscle fibroblasts, pancreatic stellate cells, and infiltrated immune cells. Tumors are typically 70-90% stroma, mostly deposited by fibroblasts and stellate cells, which causes increased intratumoral pressure, poor vascularization, and hypoxia. Therefore, drugs (chemotherapy, immunotherapy, molecular targeted therapies) delivered systemically cannot penetrate throughout the tumor (45–48). Additionally, the infiltrated immune cells generate a tumor promoting and immunosuppressive environment. For example, the immune cells of the TME are predominately regulatory T cells (T_regs_), macrophages, and myeloid-derived suppressor cells, which all work together and impede cytotoxic T cells from infiltrating, identifying, and killing tumor cells (49–53). Preclinical and clinical studies targeting the stroma and immunosuppressive pathways have had contradictory results and revealed the complexity and labyrinth-like network of interactions and pathways of the PDAC TME (54). Though progress has been made, it is essential to continue exploring and uncovering new avenues for therapeutic approaches because our current methods generate low clinical response rates. Since cancer transformation and progression are intensive mechanical processes, the molecular machinery and system responsible for cortical mechanics has the potential to be the next avenue of therapeutic approaches for PDAC.

**Figure 1:**
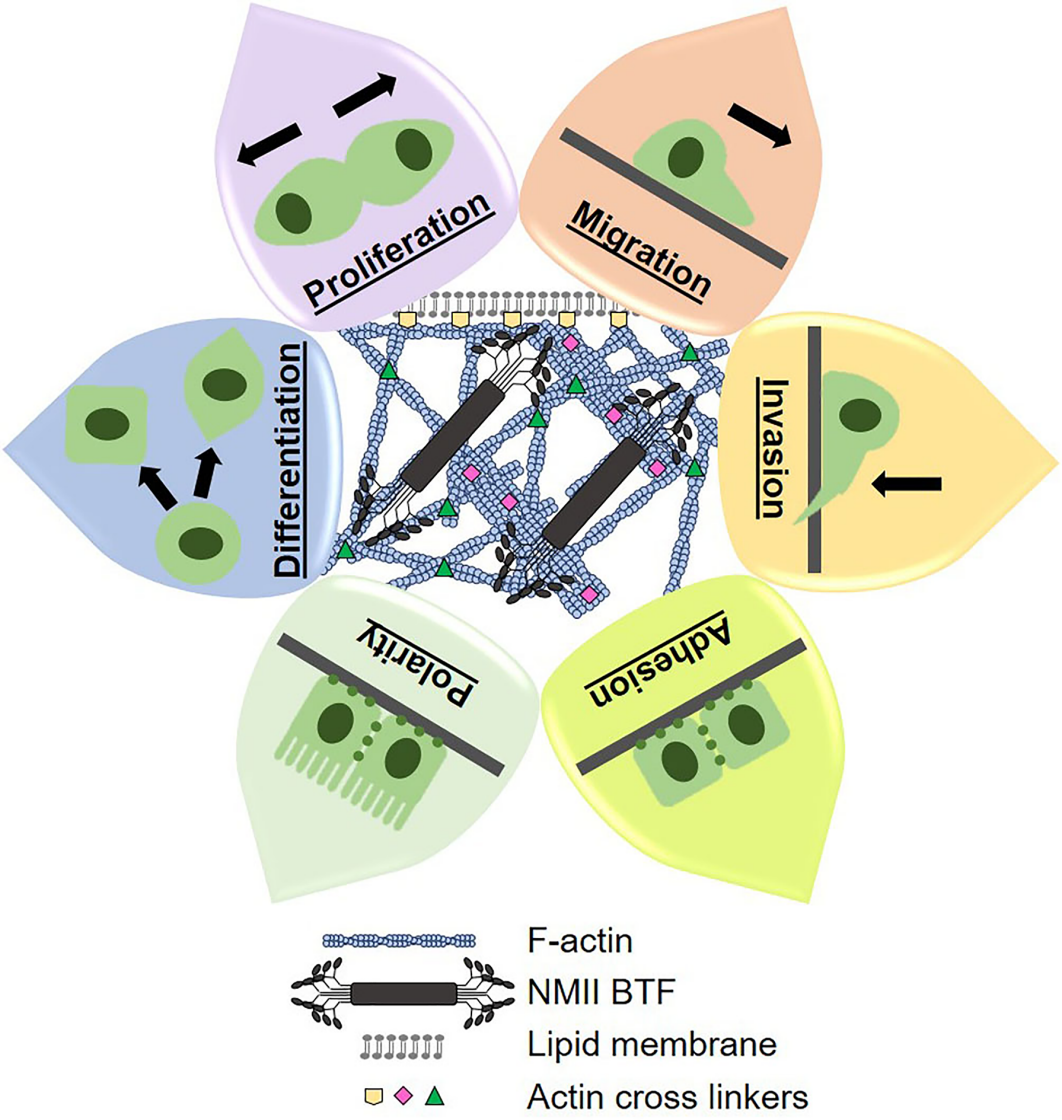
Cytoskeletal dynamics control cortical mechanics, morphogenesis, and cell behavior. The cytoskeletal components of filamentous actin (F-actin), actin crosslinking proteins, and non-muscle myosin II bipolar thick filaments (NMII BTF) dynamically assemble, polymerize, and depolymerize to generate whole cell mechanical properties and cell shape. Mechanical properties and cell shape change underlie the cell behaviors that we observe and measure. Therefore, we can manipulate cell behaviors by altering cortical mechanics and cytoskeletal dynamics, which lends great therapeutic potential.

Mechanical states of cells are defined by their underlying cytoskeletal structural and contractile machinery, which governs morphology and morphogenesis. Morphogenesis is an essential part of tumor formation and progression (*i.e.* proliferation, differentiation, polarization, migration, invasion), **Figure 1**. In general, tumor cells alter their cytoskeletal machinery to be more deformable and responsive to their changing environment. In fact, deformability has been correlated to metastatic potential and aggressiveness in many different cancer types (33, 55–57). Therefore, it is no surprise that the mechanical landscape of PDAC cells is significantly altered, which makes cortical mechanics an opportune field to explore for PDAC prognostic and therapeutic purposes (26).

Specifically, the four cytoskeletal proteins non-muscle myosin IIA (NMIIA), NMIIC, α-actinin 4, and filamin B have increased expression in PDAC patient tissues as compared to normal pancreatic ductal epithelium. The NMII paralogs are responsible for contractile forces in the cytoskeleton, and α-actinin 4 and filamin B are actin crosslinkers. In response to external stresses, each of these proteins accumulates to the site of stress to generate forces and maintain membrane-cortex integrity. We have defined this accumulation in response to physical stress as a mechanoresponse. Additionally, NMIIC is responsible for the formation of traverse actin arcs in single cells and for cortical actin belts in PDAC spheroids. Traverse actin arcs are actin bundles generated by NMII contractility and propagate toward the rear end of migrating cells as a necessary structural element driving migration. Cortical actin belts between epithelial cells generate the epithelial boundary that forms apical sides of tissues. Activating NMIIC assembly into the cytoskeleton using the small molecule 4-Hydroxyacetophenone (4-HAP) decreased *in vitro* dissemination from PDAC spheroids and *in vivo* metastasis. Specifically, 4-HAP induced cortical actin belt formation and slowed down retrograde flow of transverse actin arcs. Furthermore, 4-HAP increased cell cortical tension by activating the assembly of NMIIB and NMIIC, which also decreased migration and invasion *in vitro*. Therefore, inducing NMIIB and NMIIC assembly is a therapeutic strategy to reduce cell mobility and metastasis overall (26, 58).

Research from UCLA indicated stiffness and invasion are differentially regulated by actin and NMII proteins in accordance with disease stage in PDAC (59). The study used MIA PaCa-2 and PANC-1 cell lines derived from primary tumors and Hs766T cells derived from a metastatic site in the lymph node. Hs766T cells had higher stiffness than Panc-1 cells and were slower to round up. Furthermore, the Hs766T cells did not require NMII for invasion. Interestingly, Hs766T cells required actin filament nucleators, Arp2/3 and formin, to maintain cell stiffness and invasion. The dependence on actin filament nucleation suggests that actin polymerization is a major driving force for invasion and mechanotype (stiffness) in this metastasis-derived cell line. Invasion was not dependent on expression or activity levels in any of the cell lines. A characteristic hallmark of Arp2/3-dependent invasion is longer protrusions at the leading edge of the invading cell, which the Hs766T cell line exhibited more than the Panc-1 cells (59). This work further elucidates the altered mechanical landscape in PDAC progression and identifies potential targets specifically for metastatic PDAC cells.

Another interesting finding from an immunotherapy clinical trial implicated the NMII regulator, myosin phosphatase targeting subunit 1 (MYPT1), in PDAC. MYPT1 was originally identified through its being targeted by the immune system in a clinical trial for a cytokine-secreting whole tumor cell vaccine. An antibody response against MYPT1 in patients treated with the tumor cell vaccine correlated to a positive treatment outcome of greater than 3-years of disease-free survival. In addition to MYPT1’s elicited antibody response, its expression is highly upregulated in PDAC patient samples and in established PDAC cell lines. The function of MYPT1 overexpression and its implication in cortical mechanics has yet to be characterized in PDAC tumor cells, but its discovery through this immunotherapy clinical trial is a harbinger for the interconnectedness of the individual tumor cell cytoskeleton, immune cells of the tumor microenvironment, and therapeutic responses (60–62).

In summary, PDAC’s mechanical landscape comprising various cytoskeletal and regulatory proteins is significantly altered during transformation and the metastatic cascade. The targetability of this altered mechanical landscape using the small molecule 4-HAP demonstrated our ability to prevent metastasis, but there is much more that needs to be explored to uncover other potential therapeutics. Moreover, cortical mechanics can be used to target more than proliferation, invasion, and metastasis. While the number of studies in PDAC are still limited, when combined with research in other cancer types (12, 27), the implications and potential of targeting cell mechanics are becoming increasingly apparent *via* connections to cell stemness, differentiation, immune cell modulation, and metabolic reprogramming. The following section will provide evidence in other cancer types for such connections and illuminate cortical mechanics as a foundational property of the cell that can tie the various drivers of cancer together for more efficacious therapeutic targeting. In combination, the studies presented in this section on PDAC and the studies presented in the following section further highlight the potential of PDAC's altered mechanical landscape as being a revolutionary field for PDAC-specific therapeutic development.

## Implications for Disease Severity and Clinical Outcome

### Proliferation, Metastatic Potential, Disease Progression

Numerous reports have centered the molecular determinants of cortical mechanics in cell proliferation, migration, invasion, metastasis, and overall disease prognoses. For example, the cytoskeletal scaffolding protein anillin is extensively implicated in cancer progression and patient survival of several cancer types (*e.g.*, breast, pancreatic, colorectal, lung, gastric, liver). Generally, anillin is upregulated during cancer transformation and invasion and is associated with both positive and poor prognoses clinically depending on cell localization (63). Anillin binds to both actin filaments and NMII in the cytoskeleton and facilitates NMII localization and actomyosin contractility at the cortex of dividing cells (64, 65). Regarding migration, anillin depletion in breast cancer cells decreased *in vitro* migration and *in vivo* metastasis (66). Additionally, anillin regulates assembly of adherens and tight junctions in epithelial cells to establish the epithelial barrier. The apical actomyosin cytoskeleton physically interacts with the adhesion complexes and transfers tensile forces between cells of the epithelial barrier. Therefore, anillin being implicated in various adenocarcinoma’s disease severity and its impact on the metastatic cascade is intuitive (67–70). Additionally, the actin cross-linker and mechanoresponsive protein, filamin B, has increased expression in pancreatic cancer primary tumors and has been correlated with reduced patient survival through the analysis of publicly available data on the Oncomine and UALCAN databases (71, 72). Furthermore, filamin B is positively regulated by the pancreatic cancer-associated transcription factor MYB, which is suggested to be a potential biomarker of PDAC aggression (73, 74).

The NMII contractile protein paralogs have extensively been implicated in cancer cell behaviors and disease progression. Singh et al. found that NMIIA suppressed tumor formation, metastasis, and regulated immune cell infiltration in *in vitro* and *in vivo* melanoma models. NMIIA knockdown in B16F10 cells enhanced migration and invasion in transwell-based assays. Furthermore, subcutaneous and intravenous xenograft mouse models showed enhanced tumor cell proliferation, metastasis, and inflammatory cell infiltration in response to NMIIA knockdown (75). Specifically, they saw an increase in several oncogenes, ERK signaling, and endothelial cells in tumor sections, which suggests NMIIA is a tumor suppressor in melanoma cells. Picariello etal. (76) found that NMIIA regulates glioblastoma proliferation and invasion depending on the mechanical environment. Glioblastoma commonly invades into the surrounding brain tissue, which results in its overall aggressiveness and lethality. NMII activity was suspected to be a potential target for glioblastoma since the invasive ability of the tumor cells is dependent on NMII function (76).

Interestingly, this study discovered complete NMIIA knockout regulated cell proliferation and motility differentially depending on the stiffness of the substrate in the cells’ environment. On softer substrates, NMIIA depletion led to ERK1/2 activation, resulting in higher proliferation rates. On stiffer substrates, NMIIA depletion led to NFκB activation impacting cell survival and cell stemness. NFκB activation in response to NMIIA depletion was also observed in triple negative breast cancer cells and keratinocytes. Overall, *in vivo* experiments revealed NMIIA depletion in a mouse model led to reduced invasion, but larger tumors which hastened the overall lethality of the disease. The significant findings from this study show that NMIIA is a downstream effector that can be targeted to prevent invasion, but is also an up/mid-stream signaling component that responds to the mechanical environment and impacts disease progression. This is further complicated by the fact that each of the NMII paralogs can serve different functions within the cell (76).

The Nguyen-Ngoc et al. study found that NMIIA and NMIIB suppress breast epithelial proliferation. Using transgenic mice, they developed mammary organoids where 50-75% of the cells were NMIIA and NMIIB null, resulting in a mosaic tissue of IIA and IIB expression. Additionally, they used organoids from transgenic mice that had ubiquitous deletion of NMIIA and NMIIB expression and found that the mosaic NMIIA/IIB organoids had increased proliferation compared to the ubiquitously deleted NMIIA/IIB organoids. Furthermore, simple stimulation *via* fibroblast growth factor signaling induced hyperplasia. These results were also confirmed in an *in vivo* model. Overall, this study demonstrated NMIIA and NMIIB’s suppressive regulation of proliferation in breast epithelium (77).

The Kapoor et al. study elucidated the RhoA-ROCK-NMII pathway regulation of two distinct modes of invasion. The first being mesenchymal invasion where single cells have a spindle-like morphology and use adhesion- and matrix metalloproteinase (MMP)- dependent migration to move though tissue. The second mode uses amoeboid migration where single cells have a rounded morphology and use adhesion- and MMP-independent motility to squeeze through ECM pores. They demonstrated ovarian cancer cell lines with resistance to cisplatin used mesenchymal invasion, whereas cells resistant to paclitaxel or both drugs used amoeboid invasion. In both modes, signaling through the RhoA-ROCK2-NMII regulated invasion and, more specifically, NMIIA and NMIIB function mediated both nuclear squeezing and MMP-9 activity. Conclusively, NMIIA and NMIIB regulated both modes of invasion in ovarian cancer cells, demonstrating NMII’s role in invasion and the metastatic cascade (78).

Phosphorylation of the NMIIA heavy chain regulates breast cancer cell ability to degrade ECM and invade. NMIIA heavy chain phosphorylation regulates the myosin heavy chain’s ability to assemble into the bipolar myosin II filaments that are functional in the cytoskeleton. Phosphorylation of the heavy chain on the Ser-1943 residue promotes myosin disassembly and increased EGF-stimulated lamellipodia formation of breast cancer cells. Additionally, Ser-1943 phosphorylation is required for *in vitro* matrix degradation and increased invadopodia function, as well as increased *in vivo* metastasis. Therefore, NMIIA function regulates breast cancer cell invasion and metastasis (79). NMIIA-mediated cortical mechanics has also been implicated in colorectal cancer. Using tissue microarrays of patient tumor samples, NMIIA heavy chain overexpression positively correlated with disease progression and poor survival of patients. Overexpression of NMIIA heavy chain increased proliferation, invasion, and metastasis in both *in vitro* and *in vivo* models. At the molecular level, NMIIA heavy chain overexpression increased phosphorylation levels of both ERK and AKT, which subsequently reversed with NMIIA heavy chain knockdown (80). Altogether, the studies of the NMII paralogs in various cancer types realize NMII-modulated cortical mechanics as a foundational property of cancer cell shape control and function.

Various reports have implicated MYPT1, the NMII assembly regulator found overexpressed in PDAC (62), in the formation and prognoses of different cancers. For example, MYPT1 knockdown in HeLa cells resulted in nuclear fragmentation, nuclear compartment breakdown and genome instability (81). MYPT1 knockdown also increased histone methylation levels *via* the methyl transferase PRMT5, which is associated with transformation in hepatocellular carcinoma (82). Overexpression of the micro-RNA molecule microRNA-30d that targets MYPT1 knockdown predicted aggressive disease in prostate cancer (83). Finally, low copy number of the MYPT1 gene in colorectal cancer predicted poor clinical outcome for oxaliplatin treatment (84).

In gastric cancer, overexpression of MYPT1 presents as a tumor suppressor. MYPT1 expression in normal tissue was compared to patients’ cancer tissue and overall patient survival. MYPT1 is decreased in gastric tumors, which also correlated with poor patient survival. *In vitro*, MYPT1 overexpression inhibited proliferation, migration, and invasion. MYPT1 functions to negatively regulate NMII activity in the cytoskeleton. Therefore, gastric tumor cells decreased a myosin inhibitor to promote disease progression, ultimately increasing NMII activity and yielding NMII activity as oncogenic (85). MYPT1 was also discovered in prostate cancer as a biomarker of disease progression. Gene expression profiles of tumor cells had not adequately predicted patient outcome. Therefore, researchers investigated the TME to identify potential prognostic biomarkers using tumor microarrays of patient tumor samples paired with Aperio Imagescope software analysis. They then evaluated biomarker association to biochemical recurrence and time to biochemical recurrence. Overall, MYPT1 positively correlated with disease progression (86).

The reports mentioned thus far have focused on individual cytoskeletal elements and regulators, but there are also many reports characterizing general mechanical properties. For example, mechanical properties of human ovarian and breast cancer cell lines predicted the invasive ability of these cells. Microfluidic devices were developed to perform quantitative deformability cytometry and allowed measurement of physical phenotypes such as the cell’s elastic modulus, cell fluidity, entry time, maximum strain, and cell size. Using prediction models paired with the known phenotypes of individual cell lines, analysis indicated that the elastic modulus correlated the most with invasive ability, but the additional parameters of fluidity, entry time, and size improved the model’s predictive accuracy. Altogether, this study demonstrated the value of mechanical characteristics as biomarkers of invasion and potential targets for therapeutic intervention (87).

An *in vitro* study using osteosarcoma cells investigated the relationship between mechanical properties and metastatic potential. Researchers used a low metastatic parental line and a corresponding high metastatic line. Overall, highly metastatic cells spread less and exerted weaker forces than the line with low metastatic ability. The weaker forces of the highly metastatic cells contradict the increased traction forces of metastatic cells in other cancer types (14–16), but osteosarcoma differs in that it has mesenchymal origin and does not undergo epithelial-to-mesenchymal transition (EMT). Therefore, osteosarcoma cells do not experience similar polarity and differentiation trends as other adenocarcinomas, which exemplifies differences in mechanical properties according to differentiation and the need to characterize mechanics specific to each cancer type (88). Finally, cell and tissue stiffness are determinants in metastatic organotropism (89–92). For example, breast cancer cells subcategorized by their cytoskeletal and biophysical properties had specific metastatic preferences due to cytoskeletal adaptation ability and their corresponding gene expression patterns (93).

In summary, mechanical properties of individual cells and tissues dictate cell behaviors and overall disease outcomes, but we see a contradiction of effect for specific cytoskeletal proteins like the NMII paralogs (76–83). The contradiction of NMII function in different cancer types reveals the complexity of the cytoskeletal system and our lack of understanding of the cortical cytoskeletal role in cell function, further revealing the need for investigation and integration of cortical mechanics in the different cancer types. Yet, each of these studies points toward the cell’s mechanical landscape as a foundational system that can tie the various drivers of cancer together for more efficacious therapeutic targeting, further highlighting the role of PDAC’s altered mechanical landscape.

### Immune Cell Interaction and Immunotherapy Resistance

Cytoskeletal forces of both the TME immune cells and tumor cells regulate immune cell infiltration into tumors, immune cell polarity, the molecular interactions between cells, and the resulting anti- or pro-tumor immune response. Traction force microscopy has been used to measure Jurkat T cell force exertion during T cell receptor (TCR) activation. TCR activation is central to any adaptive immune response and mediates both antigen-specificity and cytotoxic activity against targeted cells. For the anti-tumor response, TCR activation in CD4^+^ T cells enables identification of neoantigens on tumor cells vs. normal cells and the fatal interaction of CD8^+^ T cells recognizing the tumor cells. Previous work had indicated that primary CD4^+^ T cells exerted traction forces in response to CD3 or CD28 stimulation, which is required for TCR activation and a T cell response. The cytoskeletal forces generated in the T cell are mediated by actin polymerization and NMII contractility (94–104).

A study of Jurkat T cells demonstrated that T cells spread more uniformly and exhibited larger and longer TCR signaling responses on stiffer substrates as compared to softer substrates. The differential responses of T cells based on substrate stiffness have major implications for the interactions between tumor cells and T cells and the regulation of cell stiffness and cortical tension of the individual tumor cells (105). The exact molecular mechanisms of T cell response to substrate stiffness are already being elucidated. For example, cytotoxic CD8^+^ T cells were able to kill the bulk of tumor cells, but not the undifferentiated cells with self-renewal capabilities. The undifferentiated cells are referred to as tumor repopulating cells, a subset of cancer stem cells that can be dormancy competent, and were immediately characterized as inducing PD-1 expression in CD8^+^ T cells and as being softer than the differentiated tumor cells (106–110).

Further investigation revealed cell softness prevented formation of the perforin pore in the targeted tumor cell. Perforin is released from activated CD8^+^ T cells to form a pore on the tumor cells and allow granzymes from the T cell to enter the targeted cell for apoptotic induction. NMIIA heavy chain is required for perforin pore formation because tumor cells with NMIIA heavy chain knocked down were unable to generate the actomyosin-mediated forces at the cell membrane and failed to form perforin pores. Pharmacologically increasing the stiffness of tumor repopulating cells allowed perforin pore formation and T cell-induced apoptosis in tumor cells using both *in vitro* and *in vivo* models (111). Cell softness also prevented immune synapse formation and target-induced apoptosis of natural killer cells, in addition to cytotoxic T cells. Human NK cells more effectively secreted granzymes A and B, FasL, granulysin, and IFNγ on stiffer substrates than softer substrates (112), a trend that was similarly observed in the cytotoxic T cells. Moreover, a study in cervical and colorectal cancer cell lines revealed NMII paralog-specific activity and localization induced MHCI and CD59 uptake *via* clathrin-mediated endocytosis (113).

The previous studies focused on T cell and natural killer cell interactions with tumor cells. Work has shown that the mechanical properties of tumor cells also impact the immune cells’ ability to enter and incorporate with the tumor. Specifically, knockdown of the NMIIA heavy chain in melanoma cells regulated immune cell infiltration of the TME. Using subcutaneous tumor formation and intravenous lung metastasis models, melanoma tumors with NMIIA heavy chain knocked down had increased recruitment of leukocytes (CD45^+^) and macrophages (F4/80^+^). The exact polarity and function of these cells was not further investigated, but we know immune cells in general greatly impact tumor growth, metastatic ability, and therapeutic response (75).

In summary, cortical mechanics of both tumor cells and the cells of the tumor microenvironment coordinate to facilitate immune evasion, detection, and therapeutic responses. The studies presented here indicate that targeting cell softness could enhance the tumor response to immunotherapies that induce cytotoxic T cell killing. This is pertinent to PDAC as it has repeatedly shown low response rates to anti-PD-1/PD-L1 immunotherapy. Cancer cell stemness adds to the story since mechanisms of T cell evasion have been elucidated in the tumor repopulating cells. These findings imply that we can target cortical mechanics to induce cancer cell differentiation and simultaneously prevent immune evasion. The next section will further elucidate the connection between the mechanical properties of cancer cell stemness, differentiation state, and their impact on immune cell interactions.

### Cancer Cell Stemness, Differentiation, and Disease Relapse

The case for cancer stem cells (CSC) has faced criticism due to the complexity of differentiation states and cell plasticity/adaptability, which hinder our ability to fully understand these cell populations. CSCs are characterized by self-renewal, resistance to stress, dormancy, and evading cell death (114). Clinical data from cancer patients with disease recurrence or relapse revealed the metastatic tumor cells and circulating tumor cells within these patients came from cancer cells that persisted after treatment of the primary tumor. Therefore, CSCs are suspected of being the therapy-resistant cells that are responsible for disease recurrence (115).

Molecular and genetic characterization of CSCs have further revealed subcategories such as dormancy-competent CSCs, dormancy-incompetent CSCs, and cancer-repopulating cells. Dormant cells are simply defined as cells that exit a highly proliferative state. Dormant cells are able to evade standard chemotherapies that kill highly proliferative cells due to the quiescent-like state (116, 117). Additionally, CSCs can maintain short- and long-term dormant states, which helps to explain clinical relapse after just months or years of remission (118–120). Moreover, dormancy-competent cells are able to resist immunological targeting directly and indirectly through immunosuppression. The less proliferative dormant cells are not capable of generating neoantigens like highly proliferative tumor cells, which ultimately leads to immune evasion. Dormant cells have also displayed immunosuppressive mechanisms of inhibition for T cell activation *via* overexpression of B7 homolog 1, cytotoxic T cell-induced apoptosis *via* methylation of suppressor of cytokine signaling 1, and antigen inhibition *via* decreased human leukocyte antigen expression (121–124).

We are emphasizing cancer stem cells, and the subsequent dormant and differentiated cells, in this review because interesting mechanical data has revealed a new avenue of characterization and targetability for these therapy-resistant cells. For example, research with breast, ovarian, lung, bladder, and prostate cancer cell lines revealed that dormancy competent cells entered states of dormancy and survival in response to high matrix stiffness, whereas dormancy-incompetent cells rapidly died. Both proliferative and metabolic activity were inhibited, and chemoresistance was increased in these dormant cells (125–132, 145). Ongoing research is developing ways to induce cancer cells to exit dormancy by using biomaterials. Using an agarose-silica gel-based method, breast cancer cells were able to enter dormant states and then exit by immediately regaining proliferative and migratory capabilities that were lost in the dormant state (134). Uncovering the mechanism of matrix stiffness regulating dormancy will provide new insight on how to effectively target dormant cancer cells and prevent clinical relapse. Additionally, research has focused on uncovering ways to identify cancer stem cells from the bulk tumor cells. Interestingly, cell stiffness or cell softness is a unique marker of cancer stem cells. Specifically, CSCs are significantly softer across various cancer types, and this property is mediated through stem cell factor signaling pathways (135–137).

Interestingly, NMII activity regulates the self-renewal capability in human pluripotent stem cells and mouse embryonic stem cells. For example, NMII inhibition *via* blebbistatin and RNA knockdown increased cell viability and the expression of self-renewal regulators Oct3/4 and Nanog (138). Additionally, NMIIA expression in mouse embryonic stem cells maintained E-cadherin-mediated cell adhesions. Furthermore, NMII assembly regulation *via* its phosphorylation sites is intertwined with EMT and migration. When EMT is induced *via* TGF-β stimulation in mouse epithelial cells, there was a stark increase in NMIIA Ser-1916 phosphorylation, which increased the invasive behavior of these cells. In mesenchymal stem cells, phosphorylation of NMIIA at Ser-1943 resulted in random migration on soft substrates, but dephosphorylation and subsequent assembly of NMIIA at Ser-1943 resulted when these same cells were placed on stiff substrates (139). Altogether, these results reveal the molecular determinants of cortical mechanics to actively regulate stemness.

Focusing on differentiation state, the Singh et al. study in melanoma revealed NMIIA knockdown regulated EMT. Specifically, NMIIA heavy chain knockdown increased the mesenchymal markers slug and twist and the epithelial marker E-cadherin. The final effect of the altered EMT markers is inconclusive, but demonstrates the clear connection between myosin II expression and differentiation (75). The Wang et al. study revealed NMIIA heavy chain overexpression induced EMT through upregulation of mesenchymal markers fibronectin, N-cadherin, and MMP9, and downregulation of epithelial markers ZO-1 and β-catenin. NMIIA heavy chain-mediated EMT was required for the observed aggressive phenotype (80).

Returning to the case of anillin (63, 66), this scaffolding protein impacts differentiation and stemness in addition to proliferation, migration, and invasion. Depletion of anillin in two mesenchymal-type breast cancer cell lines decreased stem cell properties. Similarly, increased anillin expression in an epithelial cell line increased stemness properties (66, 67). Interestingly, pluripotent cells of mouse embryos, *Drosophila* testes, and zebrafish retina all have higher anillin expression, while senescent human fibroblasts and cervical cancer cells have decreased anillin expression (133, 140–144). This correlation in stem-like properties is interconnected with differentiation state. For example, a decrease of anillin in lung and breast cancer cells resulted in mesenchymal-to-epithelial transition. The exact mechanisms and transcription factors responsible for connecting anillin to stemness and plasticity are currently being elucidated (63, 66).

Collectively, the studies of this section reveal the intertwined relationship between cell stemness, differentiation, and mechanical state. Additional reports exemplify the axis of regulation between matrix stiffness, cell stemness/differentiation, and NMII expression/function (146–150). Most importantly, cancer stem cells evade T cell-induced apoptosis by reducing cell stiffness and subsequently preventing perforin pore formation. Overall, these studies elucidate how we can use cytoskeletal components to identify and target cancer cell stemness and differentiation, which contribute to cancer progression, therapeutic resistance, and clinical relapse. This is relevant to PDAC because we do not have a clear consensus for cancer stem cell identification. Using PDAC cortical mechanics, we can potentially identify and target stem cells to eradicate therapeutic resistance and disease relapse caused by CSCs.

### Targeting Cytoskeletal and Metabolic Connections

One of the hallmarks of cancer cells is the metabolic reprogramming undergone to allow the cancer cells to survive in densely populated tissues with limited nutrients. The Warburg effect is a well-characterized metabolic shift in which cancer cells increase glucose uptake and glycolytic rates to increase energy production. More recent discoveries uncovered the heterogeneity of metabolic reprogramming in cancer cells to also include increases in oxidative phosphorylation and the use of alternative carbon sources (*i.e.*, glutamine, fatty acids, and serine). Various studies have revealed a metabolic regulation system between cancer cells, immune cells, and stroma and have demonstrated the targetability of these metabolic shifts in preventing disease progression (151–158). Surprisingly, studies have also revealed regulation and correlation between metabolic shifts and cytoskeletal proteins in cancer.

For example, proteomic analysis of breast cancer cells treated with doxorubicin, a chemotherapeutic agent, identified connections between the transcription of several metabolic enzymes, actin, and α-actinin. Ongoing work aims to use these proteins as treatment targets for breast cancer cells (159). Breast epithelial cells’ EMT *via* TGF-β induction can be mitigated through inhibition of phosphocholine anabolism, which subsequently changed the increased actin stress fiber formation that is associated with EMT (160). Another breast cancer study demonstrated the association of actin binding proteins with proteasome activity (161). Ezrin, a scaffolding protein that links the cytoskeleton to the plasma membrane, regulates osteosarcoma tumor progression and metastasis through alterations in lactate production and ATP-dependent oxygen consumption (162). A limitation of the aforementioned studies is the lack of a clear mechanistic pathway, but there are reports which implicate various mechanisms of regulation between metabolism and cytoskeletal components. For example, the pro-metastatic actin-binding protein Fascin that is overexpressed in lung cancer directly increases the transcription and activity of glycolytic enzymes phosphofructokinase 1 and 2 through the YAP1 transcription factor (163). Fructose-bisphosphate aldolase A (ALDOA), a glycolytic enzyme, directly interacts with actin and regulates the polymerization of actin filaments, which is crucial for migration and invasion. Furthermore, ALDOA has increased expression in renal, liver, and lung cancer cells and correlated with disease aggression and prognoses (164–167). Targeting ALDOA to prevent its interaction with actin reduced actin stress fiber content, proliferation, migration, ATP synthesis and survival in cancer cells (168, 169).

In conclusion, cytoskeletal composition and assembly level directly and indirectly regulate different metabolic pathways and can be used to reveal mechanisms and potential targets in cancer progression. Research has revealed upregulated cytoskeletal components in PDAC (26, 62). Work investigating the connection between these proteins and metabolism has yet to be published, but PDAC’s metabolic reprogramming is well documented and known to drive disease progression (Li et al., 2019). Therefore, there is a need for investigations of altered PDAC-associated metabolic pathways in connection with the upregulated cytoskeletal components.

## Future Outlook and Clinical Perspectives

The latest technology and research have revealed cancer to not only be a disease of genetics, but also a disease of epigenetics, immunology, and metabolism. Yet, therapeutic approaches thus far have targeted only one or two of the cancer-specific hallmarks, such as high proliferation rate, apoptotic evasion, immune evasion, *etc*. Unfortunately, current therapies and molecular targeting have not been a huge success for all cancers due to various reasons, including pathway redundancies and circumvention, low levels of immune cell invasion, cell plasticity, and metabolic reprogramming. For these reasons, cancers such as PDAC continue to have low clinical response rates and poor prognoses. To effectively combat cancers like PDAC, we need an innovative approach that can simultaneously impact the multiple systems driving cancer progression. Traditional combination therapies are an option, but may be limited due to only targeting a couple systems at a time, the overall drug toxicity, and the tendency of cancer cells to forget their initial disease drivers. Therefore, an ideal therapeutic approach may be to target multiple systems concurrently. The cancer cell’s mechanical system is a prospective field for target exploration, seeing as it is a foundational property of the cells and has already been tied to various systems driving the disease.

Cortical mechanics refers to the physical properties and capabilities of a cell given by the underlying cytoskeleton and the molecular properties of its various components. For example, actin polymerization and NMII contraction are largely responsible for force generation, but actin crosslinkers and mechanoresponsiveness of these proteins contribute to the load-bearing capability of cells. Ultimately, these properties of force generation and load-bearing drive cell morphogenesis and culminates into cell behaviors such as proliferation, migration, polarity, differentiation, and invasion. Therefore, these proteins are a major focus within the realm of cortical mechanics and have been implicated throughout various cancer types and studies. Thus far, cytoskeletal components and their regulation are heavily involved in invasive and metastatic potential, disease prognoses, cell fate and polarity, immune cell interactions of the tumor microenvironment, and metabolic regulation. Additionally, preclinical targeting of the cytoskeleton, specifically NMIIC assembly, has already been shown to prevent metastasis in PDAC and colorectal cancer *in vivo* models (26, 27).

Major challenges in the field of cancer cortical mechanics would need to be addressed to use this knowledge to its full potential. The first is the full elucidation of the mechanoresponse system regulating cell morphogenesis and the subsequent cell behaviors. Work thus far has focused on specific proteins in various cancer types at various stages with no indication of changes in the remaining cytoskeletal components. Therefore, there is confusing and contradicting conclusions regarding these specific proteins. For example, NMII has been characterized as both a tumor suppressor and promotor, depending on the specific paralog, cancer type, and methods used. Additionally, studies focus on expression levels of proteins with simplified binary descriptions of high vs. low. Unfortunately, many of the cytoskeletal components are filamentous and/or need to be assembled to be functional, but also must maintain a free pool of subunits in order to facilitate rapid remodeling in response to various mechanical and signaling inputs. Therefore, expression levels do not inform us of the assembled or functional fractions of these components in the cytoskeleton vs. the cytoplasm. Moreover, a concept of an optimal setpoint for the function of these proteins is becoming increasingly fundamental, which is exemplified through NMII being able to drive cancer progression, while at the same time, being tumor suppressive in other cancer types. Collectively, simply targeting these systems using inhibitors is not the correct strategy. As we see in PDAC and colorectal cancer models, the strategy of pushing the system towards over-assembly, *i.e.*, beyond the “optimal setpoint”, may block cancer progression, including metastasis, without inhibiting the protein’s tumor suppressive functions (26, 58).

Another major challenge is the siloed nature of many studies. The studies presented here have focused on cortical mechanics and just a few aspects of a cancer cell phenotypes, such as proliferation/migration, metabolism, and/or immune cell infiltration. For example, the melanoma study characterized proliferation, metastasis, and immune cell infiltration, but did not characterize the functionality or role of the immune cells in the tumor (75). We will need to understand the full integration of these concepts in order to draw conclusions on the impacts of any manipulation on the system. Finally, we need better characterization and identification of cancer stem cells within each cancer type. Undifferentiated cancer cells will remain a clinical problem due to their ability to resist various therapies, maintain migratory and proliferative capabilities, and further differentiate into tumor repopulating cells. Majority of cancer patient deaths are due to metastases, and we know metastasis results from persistent cancer cells that are able to micro- and macro-metastasize prior, during, and after therapies. Therefore, we need to develop methods to target the population of cancer stem cells in addition to the bulk tumor cells to fully address the disease long-term. Some cancers, such as PDAC, lack consensus on how to best identify these populations, creating yet another gap in our understanding. Without this understanding, we will not be able to treat clinical relapse.

**Figure 2:**
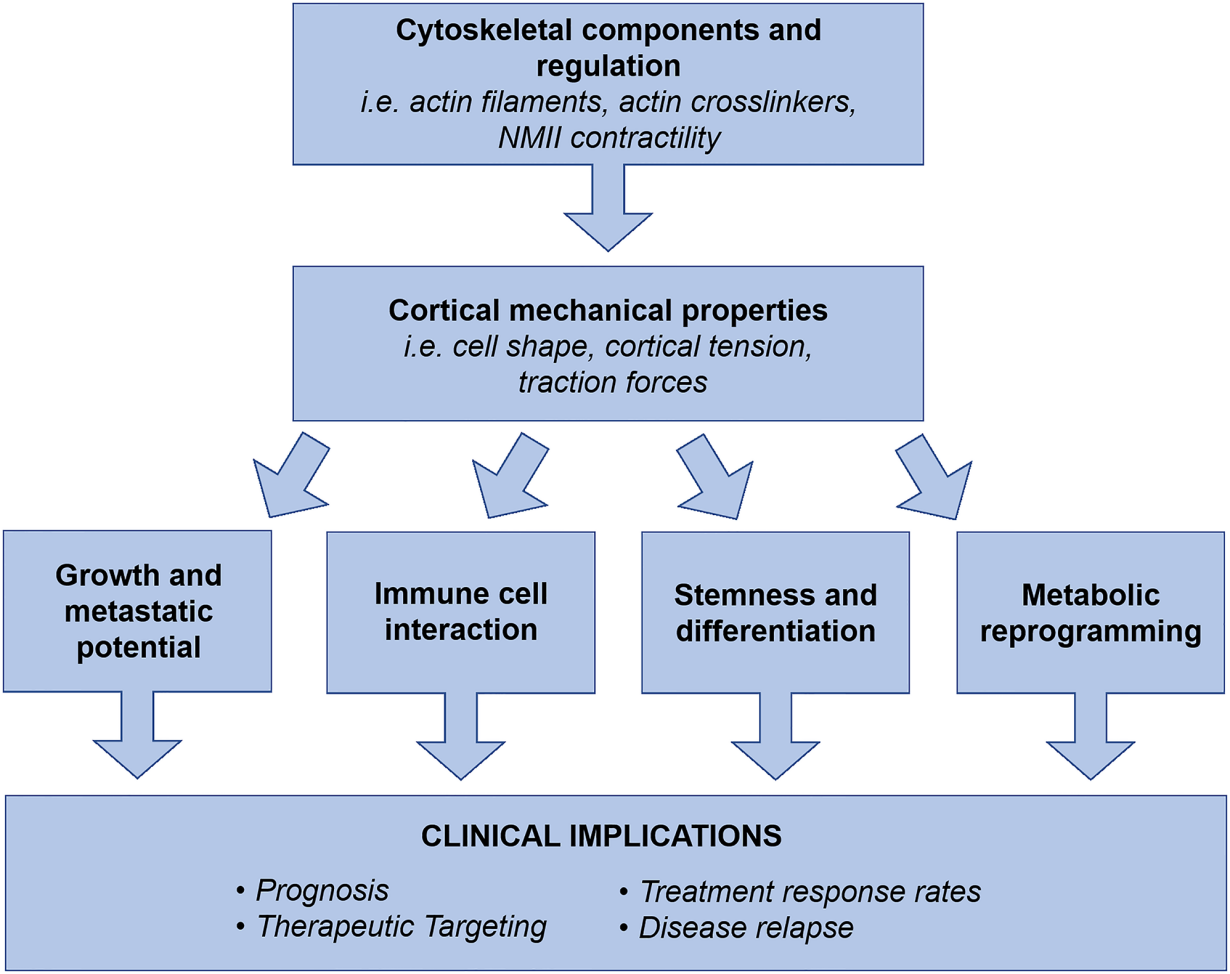
Cell cortical mechanics and its molecular drivers generate cell shape and morphogenesis, which collectively result in observed cell behaviors. The mechanical landscape and properties of cancer cells are significantly altered to drive metastasis and disease progression. Collectively, cytoskeletal components, regulators, and their resulting physical properties have been revealed as regulators of cancer cell growth and metastatic potential, TME interactions, stemness and differentiation, and metabolic reprogramming. Therefore, we propose the field of cancer cortical mechanics as a prospective area for exploration in disease prognosis, therapeutic targeting, elimination of therapeutic resistance, and prevention of clinical relapse. As a foundational system of cell behavior, cortical mechanics has the potential to concurrently address multiple drivers of disease progression, which is an ideal strategy for treating aggressive and unresponsive cancer types such as PDAC.

In summary, aggressive cancers require innovative therapeutic approaches that can concurrently target the multiple systems driving disease progression to be effective. We propose that the field of cancer cortical mechanics is a prospective area for targetability since it encompasses foundational properties of cells that interact with multiple systems (proliferation, migration, invasion, differentiation, metabolism, immune evasion) driving disease progression, **Figure 2**. Work has already uncovered PDAC’s altered mechanical landscape at the molecular level and revealed targeting NMIIC assembly can prevent metastasis, but we need further integration of this work with other fields such as metabolism, cancer stem cells, and immune cell infiltration/interaction. The integration of these systems will provide an understanding for therapeutic development that will be applicable to numerous cancer types in addition to PDAC.

## Author Contributions

SA: Conceptualization, writing (original draft), writing (review and editing), figure visualization and generation. QZ: Conceptualization, writing (review and editing). EJ: Writing (review and editing), funding acquisition, supervision. DR: Writing (review and editing), funding acquisition, supervision. RA: Writing (review and editing), funding acquisition, supervision. All authors contributed to the article and approved the submitted version.

## Funding

Our research is supported by the NIH (National Institute of General Medical Sciences Grant R01 GM66817) and the Lustgarten Foundation for Pancreatic Cancer (Project 90094641).

## Conflict of Interest

RA reports grants and personal fees from Bristol Myers Squibb, and personal fees from AstraZeneca and Merck SD. DR is currently exploring formation of a startup company. A patent has been granted on the use of 4-HAP to treat disease, and DR is an inventor on this patent. EJ reports grants from Bristol Myers Squibb and Lustgarten, as well as grants from Abmeta and Lustgarten, and personal fees from Genocea, Achilles, DragonFly, CSTONE, Candel Therapeutics, and Parker Institute.

The remaining authors declare that the research was conducted in the absence of any commercial or financial relationships that could be construed as a potential conflict of interest.

## Publisher’s Note

All claims expressed in this article are solely those of the authors and do not necessarily represent those of their affiliated organizations, or those of the publisher, the editors and the reviewers. Any product that may be evaluated in this article, or claim that may be made by its manufacturer, is not guaranteed or endorsed by the publisher.
